# Supplementary material for: MyVivarium: A cloud-based lab animal colony management application with realtime ambient sensing
Source: Comput Struct Biotechnol J. 2025 Feb 1;27:612–23. doi: 10.1016/j.csbj.2025.01.025 (PMC12621272; doi:10.1016/j.csbj.2025.01.025)
Supplement: Supplementary file 3 — Supplementary material [file mmc3.pdf]

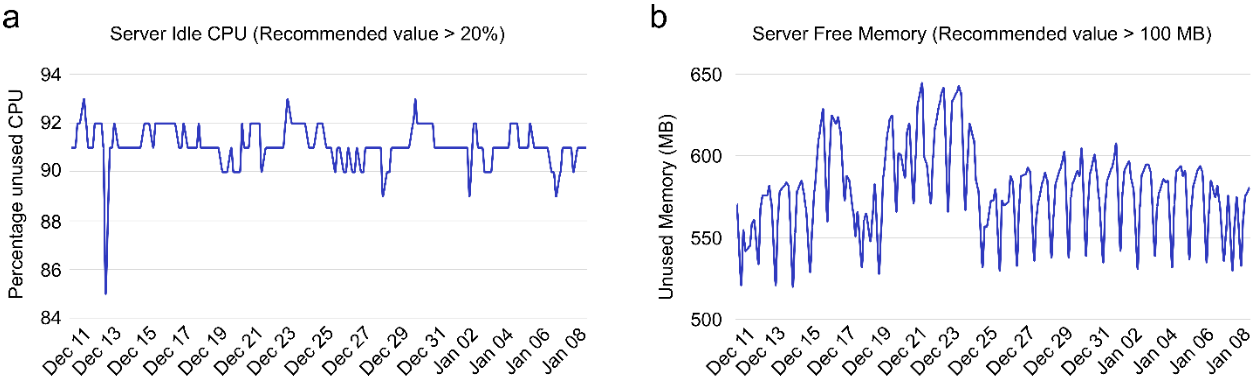

Supplemental Figure 3. Server usage and performance metrics running three MyVivarium web applications. a. Percentage of idle CPU (unused CPU) for our Cloudways server between December 8th 2024 – January 8th 2025. Note that the percentage of unused CPU is always above the recommended value of 20% **b.** Unused (free) server memory for the same time period. Note that the percentage of unused memory is always above the recommended value of 100 MB.
