# Supplementary material for: MyVivarium: A cloud-based lab animal colony management application with realtime ambient sensing
Source: Comput Struct Biotechnol J. 2025 Feb 1;27:612–23. doi: 10.1016/j.csbj.2025.01.025 (PMC12621272; doi:10.1016/j.csbj.2025.01.025)
Supplement: Supplementary file 4 — Supplementary material [file mmc4.pdf]

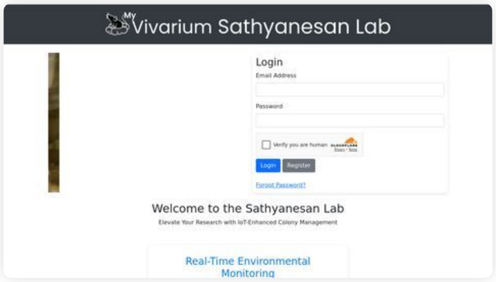

Performance Report for:

https://myvivarium.online/

Report generated: Mon, Jan 6, 2025 11:49 AM -0800
Test Server Location: Vancouver, Canada
Using: Chrome 117.0.0.0, Lighthouse 11.0.0

GTmetrix Grade	Performance	Structure	L. Contentful Paint	T. Blocking Time	C. Layout Shift
A	100%	95%	442ms	0ms	0

Top Issues

Impact	Audit	Result
Med	Use explicit width and height on image elements CLS	1 image found
Low	Allow back/forward cache restoration	2 failure reasons
Low	Eliminate render-blocking resources FCP LCP	Potential savings of 67ms
Low	Avoid chaining critical requests FCP LCP	5 chains found
Low	Avoid enormous network payloads LCP	Total size was 698KB

Focus on these audits first

These audits likely have the largest impact on your page performance.

Structure audits do not directly affect your Performance Score, but improving the audits seen here can help as a starting point for overall performance gains.

Page Details

2.6s
Fully Loaded Time

Total Page Size - 696KB

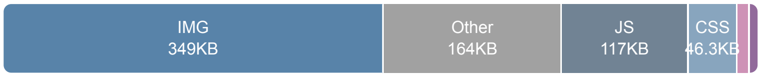

Total Page Requests - 35

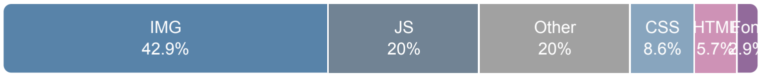

HTML JS CSS IMG Video Font Other

How does this affect me?

Modern web users have a short attention span and expect a fast and seamless website experience. Delivering that fast experience can result in more traffic, more conversions, and more happiness.

As if you didn't need more incentive, **Google use Page Speed and Page Experience (including Web Vitals) signals in their ranking algorithm.**

About GTmetrix

GTmetrix was developed as a tool for customers to easily test the performance of their webpages.

Learn more about us.

The waterfall chart displays the loading behaviour of your site in your selected browser. It can be used to discover simple issues such as 404's or more complex issues such as external resources blocking page rendering.

Sathyanesan Lab

/	200	myvivarium.online	4.98KB	159ms
**bootstrap.min.css**	200	maxcdn.bootstrapcd…	25.0KB	70ms
**bootstrap.min.css**	200	cdn.jsdelivr.net	20.4KB	18ms
**css2?family=Poppins:wg…**	200	fonts.googleapis.com	999B	44ms
**jquery.min.js**	200	ajax.googleapis.com	30.3KB	42ms
**bootstrap.min.js**	200	maxcdn.bootstrapcd…	15.3KB	83ms
**logo1.jpg**	200	myvivarium.online	117KB	158ms
**DSC_0536.webp**	200	myvivarium.online	13.7KB	177ms
**DSC_0537.webp**	200	myvivarium.online	15.4KB	316ms
**DSC_0539.webp**	200	myvivarium.online	16.8KB	185ms
**DSC_0540.webp**	200	myvivarium.online	15.7KB	191ms
**DSC_0560.webp**	200	myvivarium.online	22.2KB	201ms
**DSC_0562.webp**	200	myvivarium.online	13.2KB	206ms
**DSC_0586.webp**	200	myvivarium.online	31.3KB	207ms
**DSC_0593.webp**	200	myvivarium.online	23.8KB	206ms
**DSC_0607.webp**	200	myvivarium.online	21.4KB	206ms
**DSC_0623.webp**	200	myvivarium.online	11.2KB	205ms
**DSC_0658.webp**	200	myvivarium.online	22.5KB	206ms
**DSC_0665.webp**	200	myvivarium.online	23.7KB	207ms
**api.js**	302	challenges.cloudflar…	0	152ms
**vcd15cbe7772f49c399c6a…**	200	static.cloudflareinsig…	6.93KB	138ms
**pxiEyp8kv8JHgFVrJJfecg…**	200	fonts.gstatic.com	7.70KB	50ms
**s.js?z=JTdCJTlyZXhlY3V…**	200	myvivarium.online	4.28KB	24ms
**api.js**	200	challenges.cloudflar…	15.7KB	36ms
**auto/**	200	challenges.cloudflar…	7.39KB	46ms
**1**	200	challenges.cloudflar…	61B	19ms
**v1?ray=8fde3b6c7a722d6…**	200	challenges.cloudflar…	44.5KB	21ms
**manifest.json**	200	myvivarium.online	933B	105ms
**POST rum?**	204	myvivarium.online	0	13ms
**favicon.ico**	200	myvivarium.online	8.19KB	89ms
**android-chrome-192x192.…**	200	myvivarium.online	20.5KB	125ms
**POST ywh3HEjgEvz4GFfj…**	200	challenges.cloudflar…	113KB	97ms
**zvUR3mihxkyLL09**	401	challenges.cloudflar…	1.66KB	31ms
**C6fQjKlzdjvGWKr**	200	challenges.cloudflar…	180B	18ms
**POST ywh3HEjgEvz4GFfj…**	200	challenges.cloudflar…	19.6KB	75ms

35 Requests **696KB (1.54MB Uncompressed)** **2.6s (Onload 633ms)**

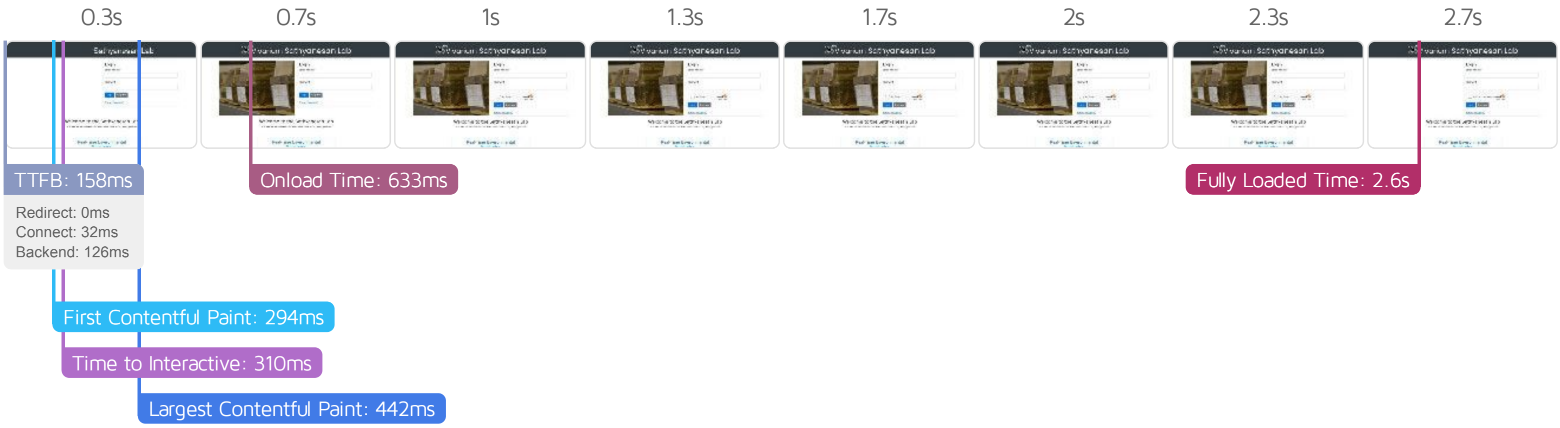

Performance Metrics

First Contentful Paint

Good - Nothing to do here

How quickly content like text or images are painted onto your page. A good user experience is 0.9s or less.

293ms

Time to Interactive

Good - Nothing to do here

How long it takes for your page to become fully interactive. A good user experience is 2.5s or less.

309ms

Speed Index

Good - Nothing to do here

How quickly the contents of your page are visibly populated. A good user experience is 1.3s or less.

374ms

Total Blocking Time

Good - Nothing to do here

How much time is blocked by scripts during your page loading process. A good user experience is 150ms or less.

0ms

Largest Contentful Paint

Good - Nothing to do here

How long it takes for the largest element of content (i.e., a hero image) to be painted on your page. A good user user experience is 1.2s or less.

442ms

Cumulative Layout Shift

Good - Nothing to do here

How much your page's layout shifts as it loads. A good user experience is a score of 0.1 or less.

0

Browser Timings

Redirect	0ms	Connect	32ms	Backend	126ms
TTFB	158ms	DOM Int.	256ms	First Paint	294ms
DOM Loaded	310ms	Onload	633ms	Fully Loaded	2.6s

IMPACT	AUDIT		
Med	**Use explicit width and height on image elements**	CLS	1 image found
Low	**Allow back/forward cache restoration**		2 failure reasons
Low	**Eliminate render-blocking resources**	FCP LCP	Potential savings of 67ms
Low	**Avoid chaining critical requests**	FCP LCP	5 chains found
Low	**Avoid enormous network payloads**	LCP	Total size was 698KB
Low	**Reduce unused CSS**	FCP LCP	Potential savings of 44.3KB
Low	**Reduce initial server response time**	FCP LCP	Root document took 125ms
Low	**Serve static assets with an efficient cache policy**		Potential savings of 7.25KB
Low	**Serve images in next-gen formats**		Potential savings of 68.7KB
Low	**Properly size images**		Potential savings of 116KB
Low	**Defer offscreen images**		Potential savings of 208KB
N/A	**Largest Contentful Paint element**	LCP	440 ms
N/A	**Reduce JavaScript execution time**	TBT	9ms spent executing JavaScript
N/A	**Avoid large layout shifts**	CLS	4 elements found
N/A	**Reduce the impact of third-party code**	TBT	Total size was 310KB
N/A	**Minimize main-thread work**	TBT	Main-thread busy for 187ms
N/A	**Avoid serving legacy JavaScript to modern browsers**	TBT	Potential savings of 56B
N/A	**Avoid an excessive DOM size**	TBT	72 elements
N/A	**User Timing marks and measures**		
